# Supplementary material for: Accelerated deprotonation with a hydroxy-silicon alkali solid for rechargeable zinc-air batteries
Source: Nat Commun. 2023 Nov 1;14:6968. doi: 10.1038/s41467-023-42728-y (PMC10618233; doi:10.1038/s41467-023-42728-y)
Supplement: Supplementary file 1 — Supplementary Information [file 41467_2023_42728_MOESM1_ESM.pdf]

Accelerated deprotonation with a hydroxy-silicon alkali solid for rechargeable zinc-air batteries

Wang *et al.*

Supplementary Note 1. DFT Calculation method.

Current first-principle DFT calculations are performed by the VASP (VASP) and PAW (PAW) methods. The exchange functional employs the generalized gradient approximation (GGA) of the Perdew-Burke-Ernzerhof (PBE) functional. All calculations are spin-polarized. The diffusion energy of the plane wave substrate is set to 450 eV, and the per-atom force below 0.03 eV/Å is set as a convergence criterion. Brillouin zone integration was performed using 3x2x1 k-point sampling. An energy threshold of 10^{-4} eV was adopted for the self-consistent calculation. The effect of van der Waals was investigated using the DFT-D3 method. Add 12 Å vacuums in the z direction to prevent interaction between periodic structures.

In an alkaline environment, OER can be performed in the following four basic steps:

$$OH^- + * \rightarrow *OH + e^- \quad (1)$$

$$*OH + OH^- \rightarrow *O + H_2O + e^- \quad (2)$$

$$*O + OH^- \rightarrow *OOH + e^- \quad (3)$$

$$*OOH + OH^- \rightarrow * + O_2 + H_2O + e^- \quad (4)$$

The * represents the reaction site on the surface of the catalyst. According to the above mechanism, under the action of OER of a given substance, the free energy of the three intermediate states is the key to determine the activity of OER. To calculate the free energy of OER, a hydrogen electrode model63 is used.

The free energy of the OER/ORR step is calculated using equation (5):

$$\Delta G = \Delta E_{DFT} + \Delta E_{ZPE} - T\Delta S \quad (5)$$

where ΔE_{DFT} is the DFT energy difference, and ΔE_{ZPE} and $T\Delta S$ obtained from the vibration analysis.

Supplementary Note 2. Fabrication of Zn-Air Batteries

Home-made Zn-Air batteries were assembled with air cathode, metal anode (high purity Zn foil), and electrolyte (6 M KOH + 0.2 M $ZnCl_2$ aqueous solution). To prepare the air cathode, the as-prepared catalyst and acetylene black with a mass ratio of 2:1 were dispersed in the mixed solution which containing isopropanol and Nafion (5%

commercially available solution) with volume ratio of 9:1 by ultrasonication for 30 min. The ink-liked solution was then drop-casted on the surface of a gas diffusion layer with the catalyst loading of 1 mg cm^{-2}. For comparison, Pt/C and mixture of Pt/C and RuO_2 (mass ratio of 1:1) were also prepared with above procedure for pristine and rechargeable Zn-air batteries respectively. All batteries were tested at ambient atmosphere and room temperature of 25 °C.

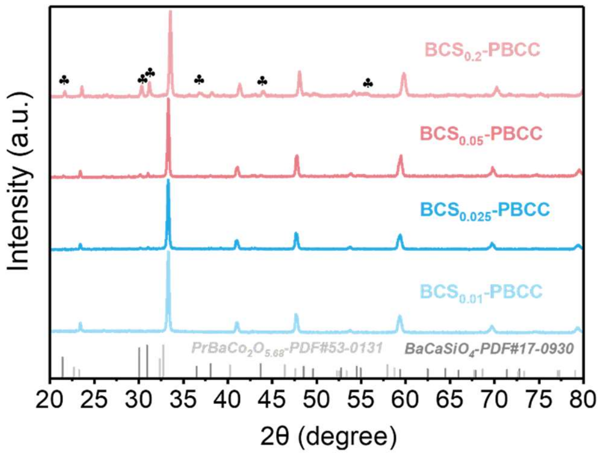

Fig. S1. The XRD patterns of the Si-incorporated $BCS_{0.2}$-PBCC, $BCS_{0.05}$-PBCC, $BCS_{0.025}$-PBCC and $BCS_{0.01}$-PBCC perovskites.

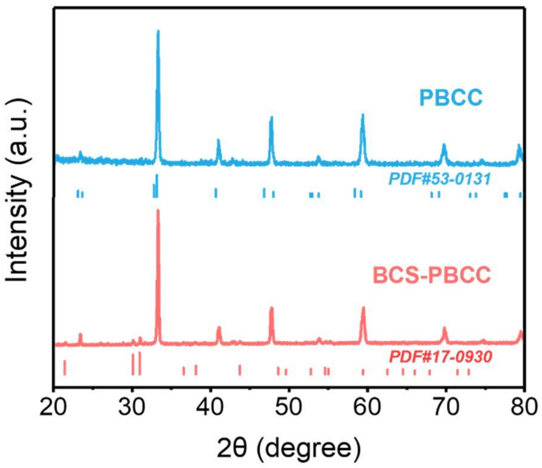

Fig. S2. The XRD patterns of the pristine PBCC catalyst and BCS-PBCC catalyst.

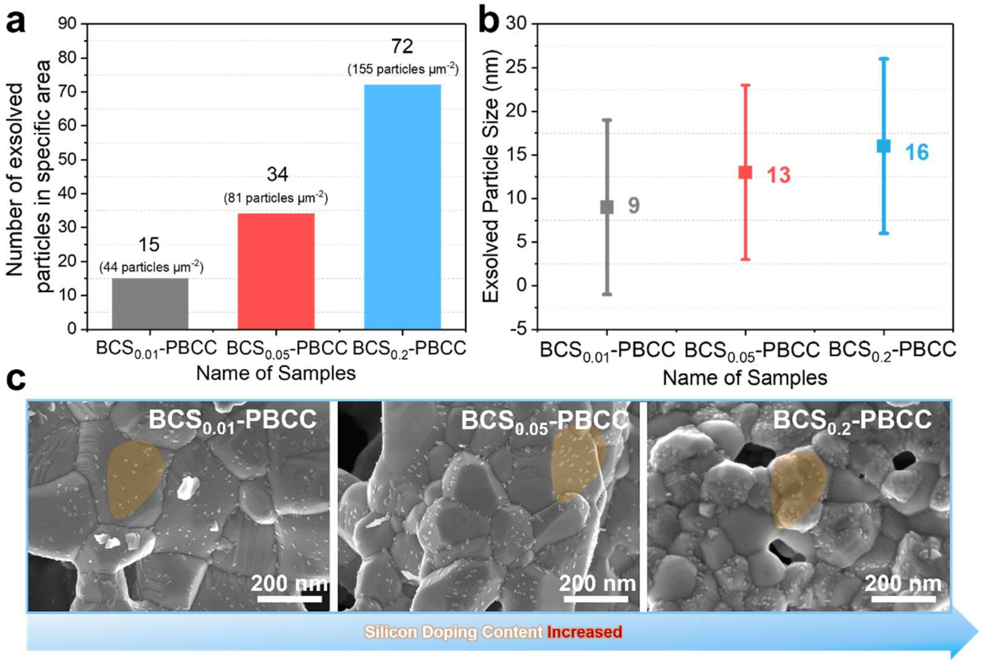

Fig. S3. Scanning electron microscopy images and the correlation between the amount of silicon doping (1%~20%) and the particle population/size. **a** ImageJ software was used to quantify the number of particles. The exsolution number of BCS nanoparticles on the PBCC surface follows the order: $BCS_{0.01}$-PBCC (44 particles/mm^2) < $BCS_{0.05}$-PBCC (81 particles/mm^2) < $BCS_{0.2}$-PBCC (155 particles/mm^2). **b** Exsolved particle size of $BCS_{0.01}$-PBCC, $BCS_{0.05}$-PBCC, and $BCS_{0.2}$-PBCC. **c** Scanning electron microscopy images of surface silicon particles as a function of increasing doping.

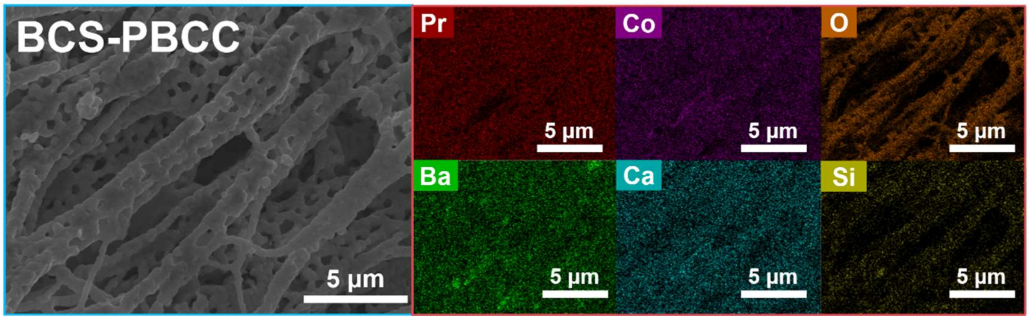

Fig. S4. The corresponding EDX element mapping of BCS-PBCC and highlight images of each element.

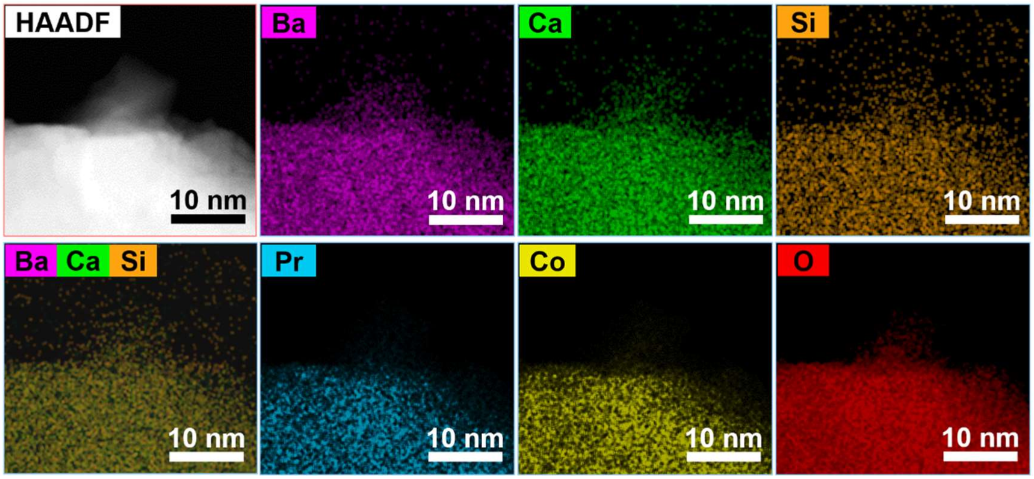

Fig. S5. HRTEM images of surface regions of BCS-PBCC, and EDS elemental map of Pr, Ba, Ca, Co, Si, O.

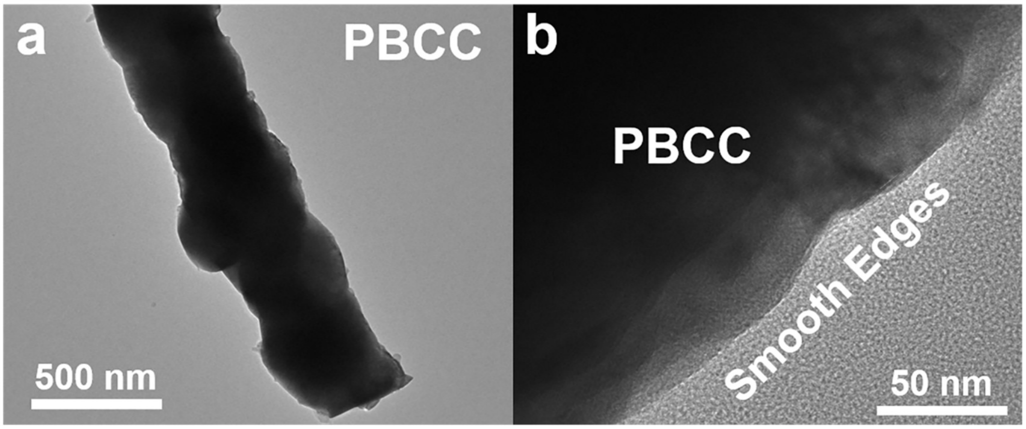

Fig. S6. a HR-TEM image of the perovskite oxide in PBCC. **b** Local magnified image showing smooth edges.

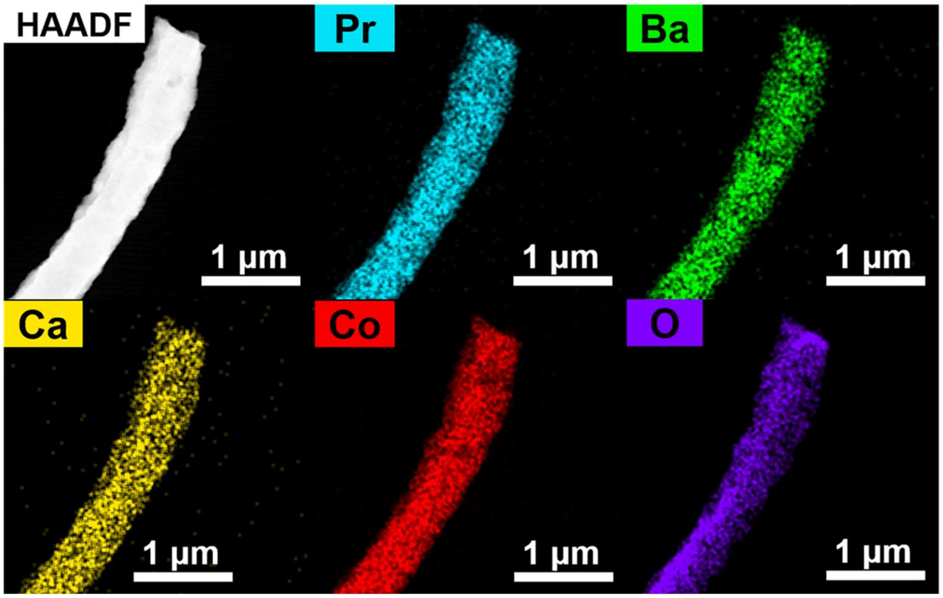

Fig. S7. HRTEM images of surface regions of PBCC, and EDS elemental map of Pr, Ba, Ca, Co, O.

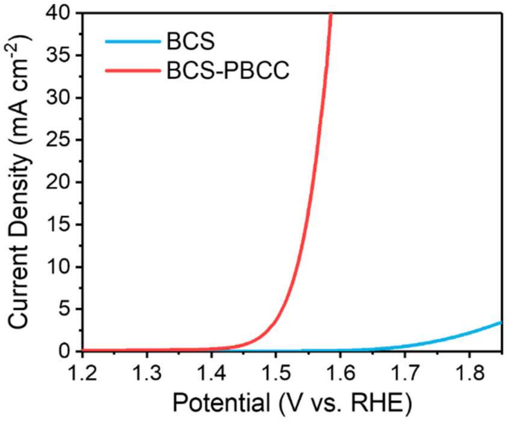

Fig. S8. Polarization curves of BCS-PBCC and BCS catalysts in an O_2-saturated 1 M KOH solution.

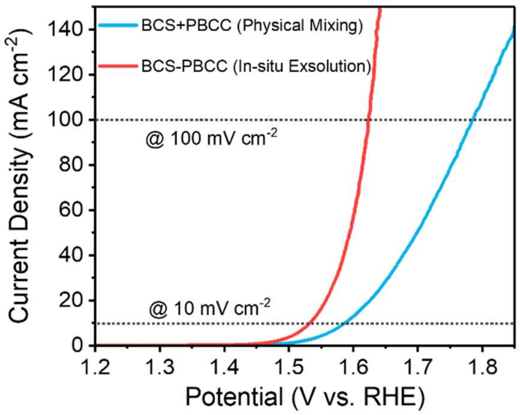

Fig. S9. Polarization curves of physically mixed BCS+PBCC and in situ ex-solved BCS-PBCC catalysts in O_2-saturated 1 M KOH solution.

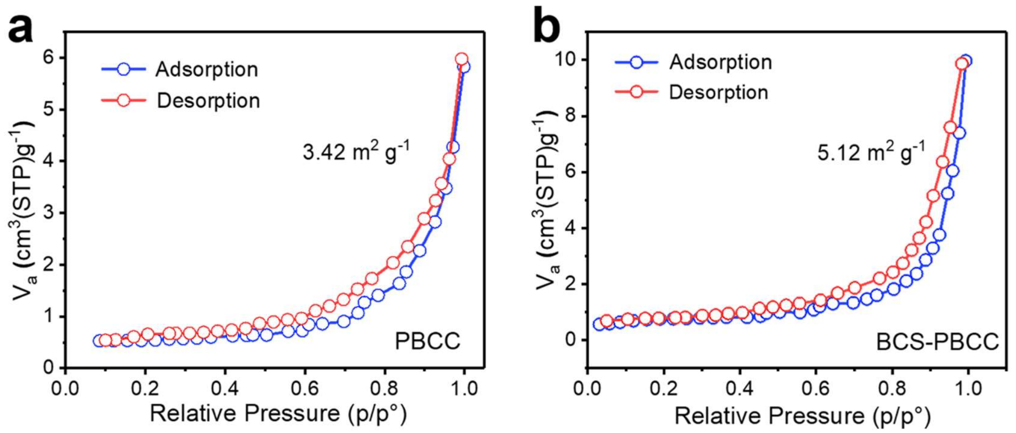

Fig. S10. Nitrogen adsorption-desorption isotherm curves of **a** PBCC and **b** BCS-PBCC samples.

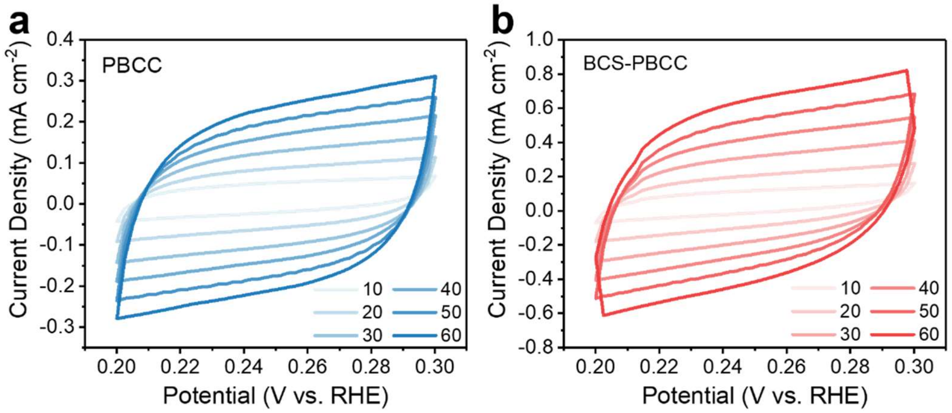

Fig. S11. ECSA estimation determined from C_{dl}. The C_{dl} obtained from the CV method is expected to be linearly proportional to the ECSA. CV measurements in a non-faradic current region (0.2−0.3 V vs. RHE, no $iR_{corrected}$) at scan rates of 10, 20, 30, 40, 50 and 60 mV s^{-1} of **a** PBCC and **b** BCS-PBCC.

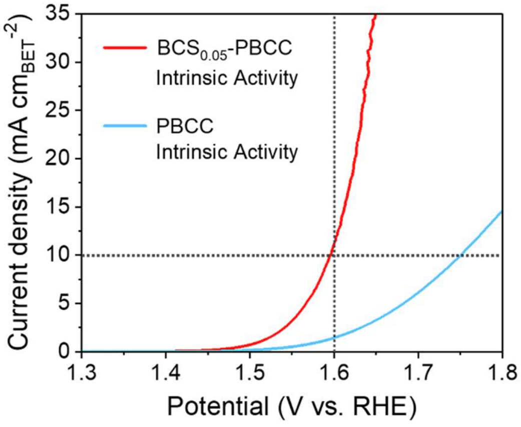

Fig. S12. The intrinsic activity of BCS-PBCC and PBCC.

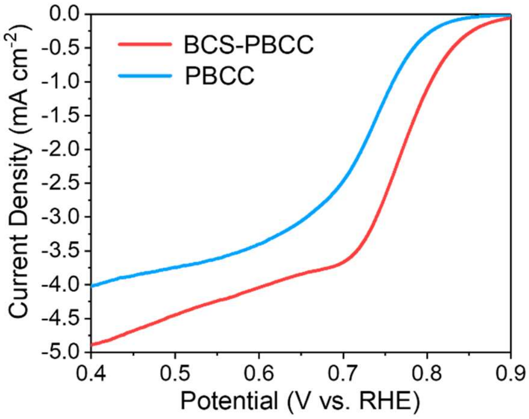

Fig. S13. LSV curves of ORR for PBCC and BCS-PBCC.

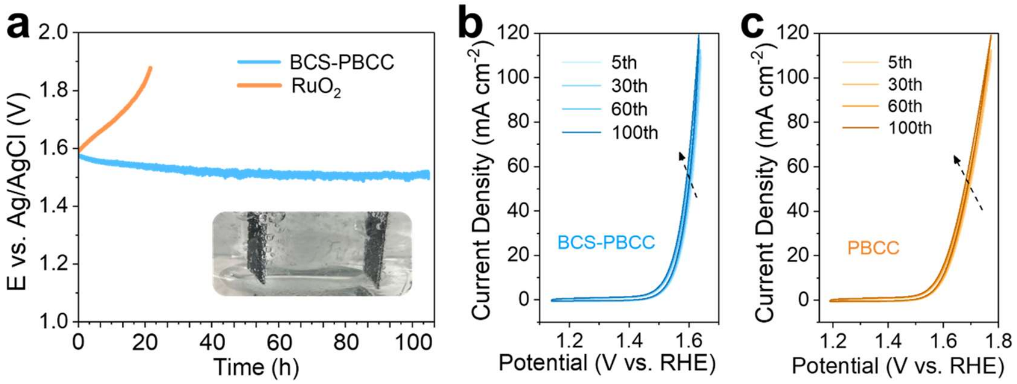

Fig. S14. a Chronopotentiometry curve of water electrolysis using BCS-PBCC and RuO_2 as the anode at a constant current density of 10 mA cm^{-2} in 1 M KOH. Inset: a digital image of the electrode in the chronopotentiometry test. Select CV curves of **b** BCS-PBCC and **c** PBCC in O_2-saturated 1 M KOH over 100 cycles at a 20 mV s^{-1} scan rate.

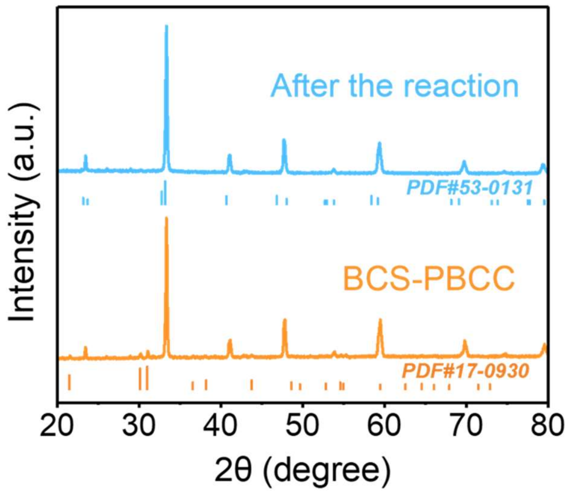

Fig. S15. XRD pattern of BCS-PBCC after ADT.

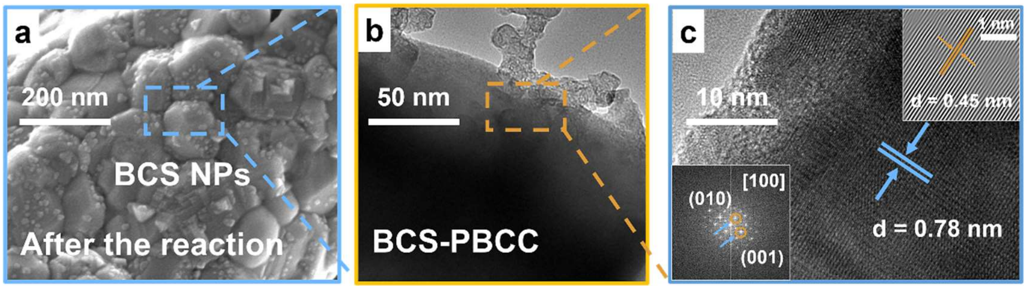

Fig. S16. a SEM image after reaction and **b** HRTEM image of BCS-PBCC after ADT. **c** corresponding FFT pattern, inset: the corresponding FFT pattern and corresponding IFFT images.

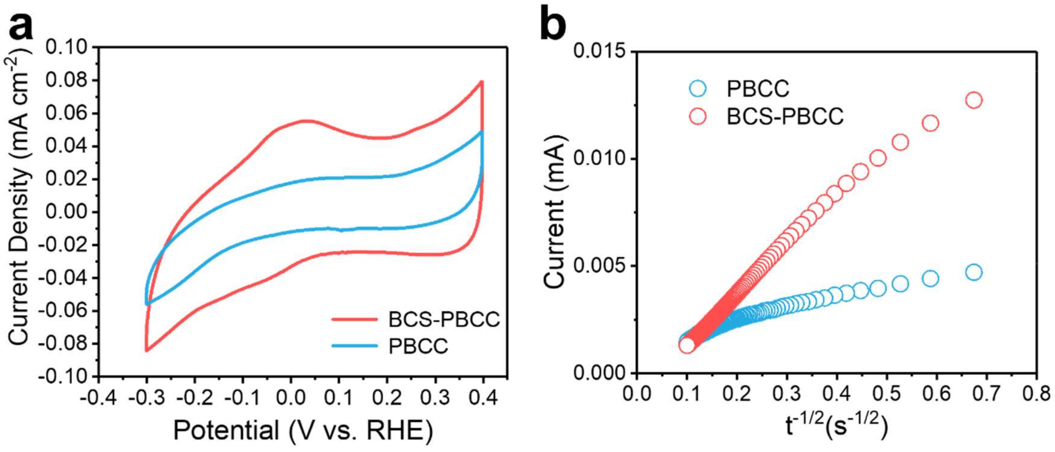

Fig. S17. a CV curves of PBCC and BCS-PBCC in Ar-saturated 6 M KOH, where redox peaks indicate the electrochemical oxygen intercalation/deintercalation. **b** shows the chronoamperometry data (i vs. $t^{-1/2}$) used for the calculation of oxygen ion diffusion coefficients.

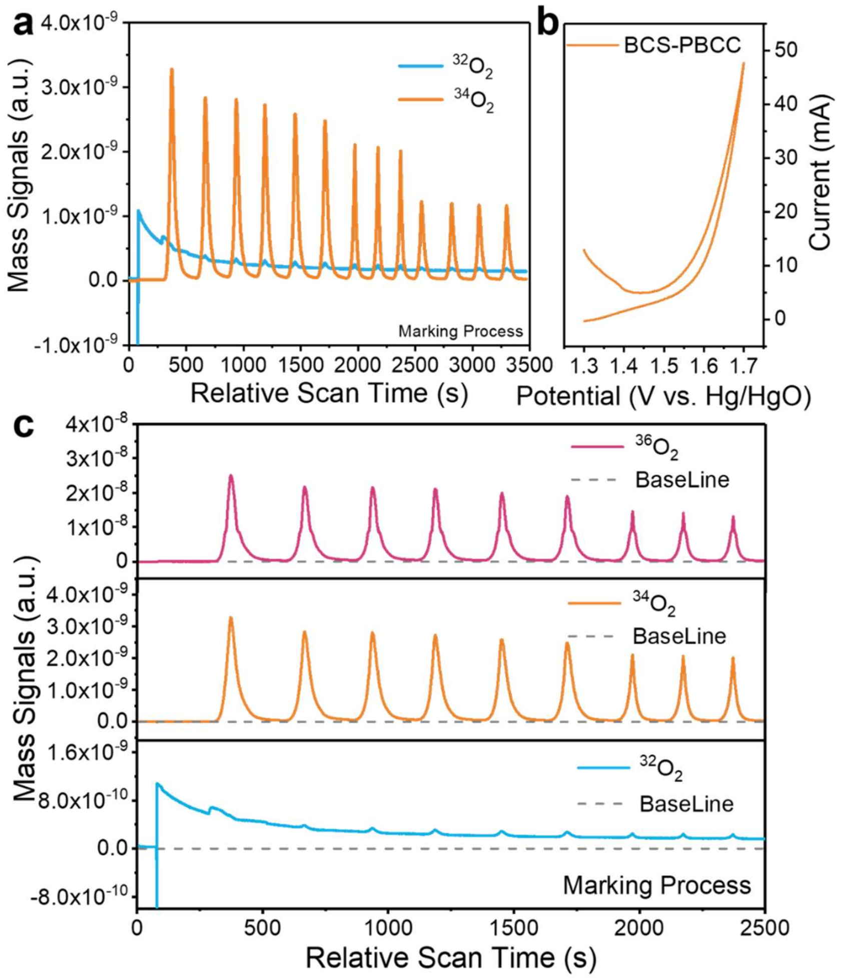

Fig. S18. a DEMS signals of $^{32}O_2$ ($^{16}O^{16}O$) and $^{34}O_2$ ($^{16}O^{18}O$) from the reaction products for ^{18}O-labeled BCS-PBCC catalyst in $H_2{}^{18}O$ aqueous KOH electrolyte and **b** corresponding CV cycles. The mass spectroscopy signals are baseline subtracted. **c** DEMS signals of $^{34}O_2$ and $^{36}O_2$ from the reaction products cycled in $H_2{}^{18}O$ aqueous sulfuric acid electrolyte.

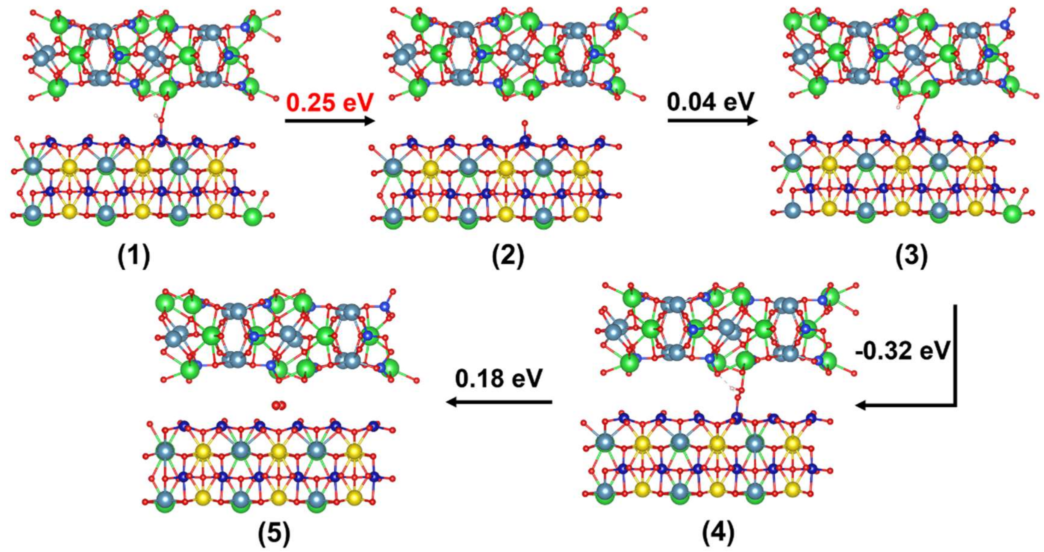

Fig. S19. Structural schematic diagram and energy barrier change of BCS-PBCC reaction in AEM pathway during OER. Color representation: Pr (yellow), Ba (green), Ca (grey), Co (navy blue), Si (light blue), O (red), H (pink).

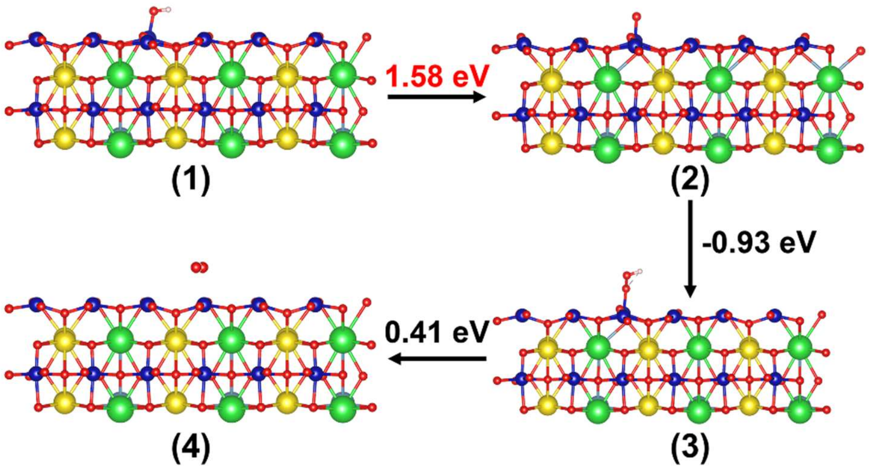

Fig. S20. Structural schematic diagram and energy barrier change of PBCC reaction in AEM pathway during OER. Color representation: Pr (yellow), Ba (green), Ca (grey), Co (navy blue), Si (light blue), O (red), H (pink).

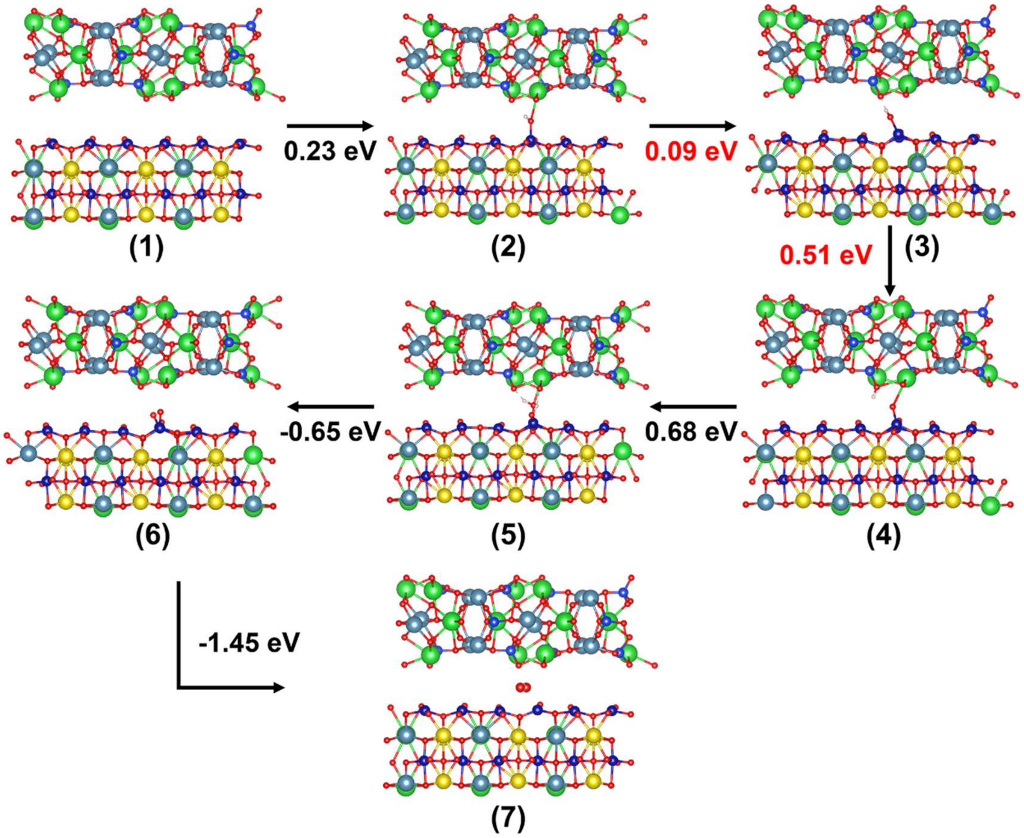

Fig. S21. Structural schematic diagram and energy barrier change of BCS-PBCC reaction in LOM pathway during OER. Color representation: Pr (yellow), Ba (green), Ca (grey), Co (navy blue), Si (light blue), O (red), H (pink).

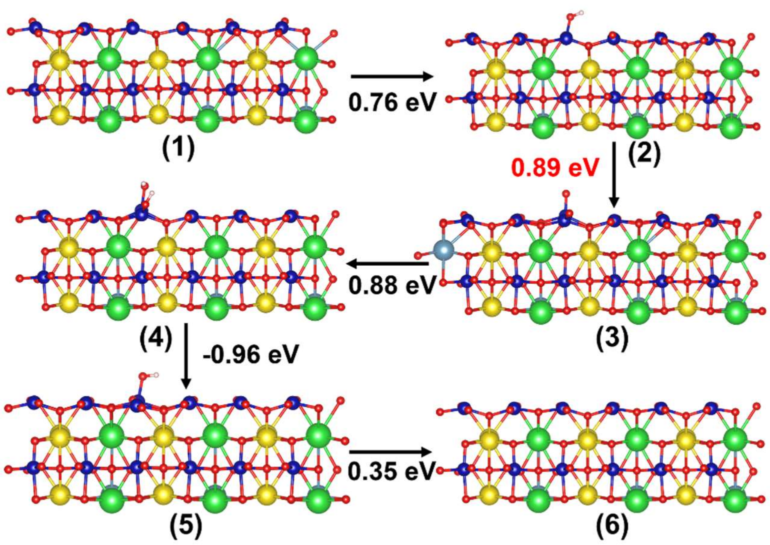

Fig. S22. Structural schematic diagram and energy barrier change of PBCC reaction in LOM pathway during OER. Color representation: Pr (yellow), Ba (green), Ca (grey), Co (navy blue), Si (light blue), O (red), H (pink).

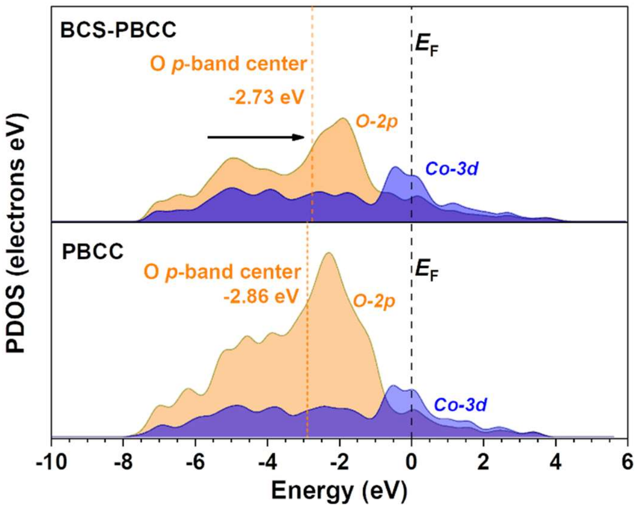

Fig. S23. Projected density of states (E_F: Fermi level, O *2p* band center).

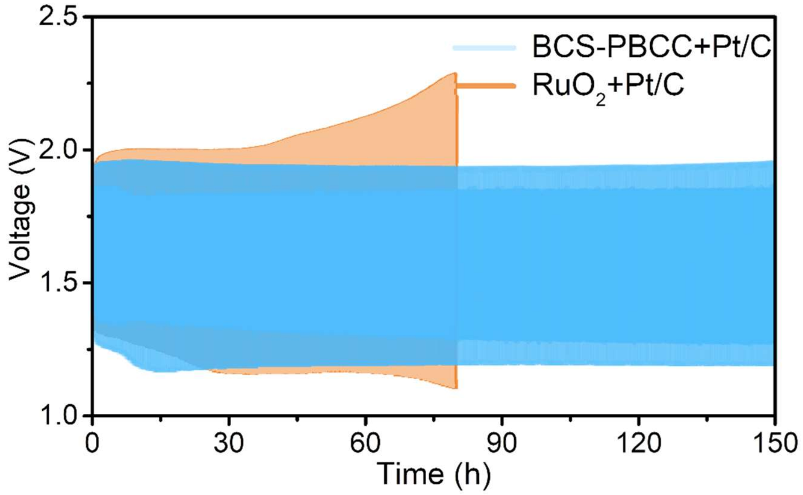

Fig. S24. Galvanostatic charge/discharge test at 5 mA cm^{-2} for Zn-air batteries with BCS-PBCC + Pt/C and RuO_2 + Pt/C as air cathode.

Supplementary Table 1. Rietveld refinement results of XRD patterns of Si-incorporated perovskites.

Perovskite	$PrBa_{0.5}Ca_{0.5}Co_2O5_{5+\delta}$	$BaCaSiO_4$
**Phase Content**	95.855	4.145
**Space Group**	P b n m	P 63/mmc
**a (Å)**	5.41775(83)	5.7812(13)
**b(Å)**	5.38692(86)	5.7812(13)
**c(Å)**	7.6209(11)	7.3958(27)
α (°)	90	90
β (°)	90	90
γ (°)	90	120
**Volume(Å^3)**	222.417(59)	214.07(12)

Supplementary Table 2. Summary of OER activity in alkaline media for various representative state-of-the-art catalysts.

Electrocatalyst	Over-potential [mV vs RHE]	Tafel slope [mV dec^{-1}]	Electrolyte	Loading [mg cm^{-2}]	Electrode	Ref.
BCS-PBCC	300	49	1.0 M KOH	0.202	GC	(This Work)
$LaCoO_3$	470	180	1.0 M KOH	/	GC	1
$LaFeO_3$	420	62	1.0 M KOH	0.232	GC	2
$SmBa_{0.5}Sr_{0.5}Co_2O_{6-\delta}$	370	46	0.1 M KOH	0.5	GC	3
$La_{0.6}Sr_{0.4}CoO_3$	426	/	1.0 M KOH	0.212	GC	4
$La_{0.2}Sr_{0.8}FeO_{3-\delta}$	370	60	0.1 M KOH	0.232	GC	5
$CaCu_3Ti_4O_{12}$	/	46	0.1 M KOH	0.096	GC	6
$Pr_{0.5}Ba_{0.5}CoO_{3-\delta}$	440	82	0.1 M KOH	0.39	GC	7
$PrBa_{0.25}Sr_{0.75}Co_2O_{5.95}$	290	75.8	0.1 M KOH	0.545	GC	8
$La_{0.6}Sr_{0.4}Co_{0.8}Fe_{0.2}O_3$	353	63	1.0 M KOH	0.245	GC	9
$La_{0.8}Sr_{0.2}Co_{0.8}Fe_{0.2}O_{3-\delta}$	248	51	1.0 M KOH	0.245	GC	9
$LaSr_3Co_{1.5}Fe_{1.5}O_{10-\delta}$	388	84	0.1 M KOH	0.255	GC	10
$La_{0.5}Sr_{0.5}Ni_{0.4}Fe_{0.6}O_{3-\delta}$	330	76	1.0 M KOH	0.4	GC	11
$La_{1.5}Sr_{0.5}NiMn_{0.5}Ru_{0.5}O_6$	430	/	0.1 M KOH	0.255	GC	12
$CaLaScRuO_{6+\delta}$	478	84	1.0 M KOH	0.708	GC	13
$Sr_{0.9}Na_{0.1}RuO_3$	170	/	0.1 M $HClO_4$	0.510	GC	14
$Sr_2Fe_{0.8}Co_{0.2}Mo_{0.65}Ni_{0.35}O_{6-\delta}$	310	56	0.1 M KOH	0.232	GC	15
$PrBa_{0.5}Sr_{0.5}Co_{1.5}Fe_{0.5}O_{5-\delta}$	370	67	0.1 M KOH	0.202	GC	16
$PrBa_{0.85}Ca_{0.15}MnFeO_{5+\delta}$	400	88	0.1 M KOH	0.2806	GC	17
$LaNi_{0.85}Mg_{0.15}O_3$	450	95	0.1 M KOH	0.153	GC	18

La_2NiMnO_6	370	58	1.0 M KOH	/	GC	[19]
$LaCo_{0.9}Ni_{0.1}O_3$	650	73	0.1 M KOH	/	GC	[20]
$LaCo_{0.8}V_{0.2}O_3$	306	40	1.0 M KOH	/	GC	[21]
$SrCo_{0.4}Fe_{0.2}W_{0.4}O_{3-\delta}$	296	50	0.1 M KOH	0.232	GC	[22]
$BaZr_xFe_{1-x}O_{3-\delta}$	412	97	0.1 M KOH	1	GC	[23]
$Ba_2CoMo_{0.5}Nb_{0.5}O_{6-\delta}$	435	77	0.1 M KOH	0.232	GC	[24]
$BaCo_{0.7}Fe_{0.2}Sn_{0.1}O_{3-\delta}$	450	69	0.1 M KOH	0.232	GC	[25]
$BaCo_{0.5-x}Fe_{0.5-x}Zr_xY_xO3-\delta$	360	69	0.1 M KOH	0.255	GC	[26]
$SrNb_{0.1}Co_{0.7}Fe_{0.2}O_{3-\delta}$	420	76	0.1 M KOH	0.232	GC	[27]
$BaCo_{0.8-x}Fe_xZr_{0.1}Y_{0.1}O_3$	420	83	0.1 M KOH	0.232	GC	[28]
$Sr_2Fe_{0.8}Co_{0.2}Mo_{0.6}Co_{0.4}O_{6-\delta}$	345	60	0.1 M KOH	0.232	GC	[29]
$SrM_{0.9}Ti_{0.1}O_{3-\delta}$	510	88	0.1 M KOH	0.32	GC	[30]
Co-doped 6H-$SrIrO_3$	235	52	0.1 M $HClO_4$	0.45	GC	[31]
$Ba_4PrIr_3O_{12}$	278	/	0.1 M $HClO_4$	0.562	GC	[32]
$Sr_2FeIr(V)O_6$	300	/	0.1 M $HClO_4$	0.25	GC	[33]
$SrCo_{0.9}Ir_{0.1}O_{3-\delta}$	300	/	0.1 M $HClO_4$	0.25	GC	[34]
$CaCu_3Ru_4O_{12}$	171	40	0.5 M H_2SO_4	0.25	GC	[35]
$PrBa_{0.5}Sr_{0.5}Co_{1.5}Fe_{0.5}O_{5+\delta}$	359	56	0.1 M KOH	0.2	GC	[36]
$SrCo_{0.95}P_{0.05}O_{3-\delta}$	480	84	0.1 M KOH	0.232	GC	[37]
$SrCo_{0.85}Fe_{0.1}P_{0.05}O_{3-\delta}$	290	52	1.0 M KOH	0.3	Ni foam	[38]
F-BSCF	280	/	1.0 M KOH	0.255	GC	[39]
$La_{0.5}Ba_{0.25}Sr_{0.25}CoO_{2.9-\delta}F_{0.1}$	/	44	1.0 M KOH	0.274	GC	[40]
**Proton Acceptor-Oxide Composite Catalyst**						

PO_4-$PrBa_{0.5}Ca_{0.5}Co_2O_{5+\delta}$	290	51	0.1 M KOH	0.202	GC	[41]
$Sr(Co_{0.8}Fe_{0.2})_{0.7}B_{0.3}O_{3-\delta}$	340	58	0.1 M KOH	0.232	GC	[42]
$MoS_2@SrCoO_{3-\delta}$	351	37	0.1M KOH	/	GC	[43]

References

1. Tong, Y. *et al.* Spin-State Regulation of Perovskite Cobaltite to Realize Enhanced Oxygen Evolution Activity. *Chem* **3**, 812–821 (2017).
2. Dai, J. *et al.* Enabling High and Stable Electrocatalytic Activity of Iron-Based Perovskite Oxides for Water Splitting by Combined Bulk Doping and Morphology Designing. *Adv. Mater. Interfaces* **6**, 21576135 (2019).
3. Zhang, H. *et al.* Morphology, crystal structure and electronic state one-step co-tuning strategy towards developing superior perovskite electrocatalysts for water oxidation. *J. Mater. Chem. A* **7**, 19228–19233 (2019).
4. Zhang, L. *et al.* Integrating the cationic engineering and hollow structure engineering into perovskites oxides for efficient and stable electrocatalytic oxygen evolution. *Electrochim. Acta* **327**, 135033 (2019).
5. She, S. *et al.* Systematic Study of Oxygen Evolution Activity and Stability on $La_{1-x}Sr_xFeO_{3-\delta}$ Perovskite Electrocatalysts in Alkaline Media. *ACS Appl. Mater. Interfaces* **10**, 11715–11721 (2018).
6. Kushwaha, H. S., Halder, A., Thomas, P. & Vaish, R. $CaCu_3Ti_4O_{12}$: A Bifunctional Perovskite Electrocatalyst for Oxygen Evolution and Reduction Reaction in Alkaline Medium. *Electrochim. Acta* **252**, 532–540 (2017).
7. He, D., He, G., Jiang, H., Chen, Z. & Huang, M. Enhanced durability and activity of the perovskite electrocatalyst $Pr_{0.5}Ba_{0.5}CoO_{3-\delta}$ by Ca doping for the oxygen evolution reaction at room temperature. *Chem. Commun.* **53**, 5132–5135 (2017).
8. Wu, Z. *et al.* Effect of Sr doping on the electrochemical properties of bi-functional oxygen electrode $PrBa_{1-x}Sr_xCo_2O_{5+\delta}$. *J. Power Sources* **334**, 86–93 (2016).
9. Zhao, C. *et al.* Surface Reconstruction of $La_{0.8}Sr_{0.2}Co_{0.8}Fe_{0.2}O_{3-\delta}$ for Superimposed OER Performance. *ACS Appl. Mater. Interfaces* **11**, 47858–47867 (2019).
10. Liu, S., Luo, H., Li, Y., Liu, Q. & Luo, J. L. Structure-engineered electrocatalyst enables highly active and stable oxygen evolution reaction over layered perovskite $LaSr_3Co_{1.5}Fe_{1.5}O_{10-\delta}$. *Nano Energy* **40**, 115–121 (2017).
11. Wang, C. C., Cheng, Y., Ianni, E., Jiang, S. P. & Lin, B. A highly active and stable $La_{0.5}Sr_{0.5}Ni_{0.4}Fe_{0.6}O_{3-\delta}$ perovskite electrocatalyst for oxygen evolution reaction in alkaline media. *Electrochim. Acta* **246**, 997–1003 (2017).
12. Retuerto, M. *et al.* $La_{1.5}Sr_{0.5}NiMn_{0.5}Ru_{0.5}O_6$ Double Perovskite with Enhanced ORR/OER Bifunctional Catalytic Activity. *ACS Appl. Mater. Interfaces* **11**, 21454–21464 (2019).
13. Kumar, N. *et al.* Investigation of New B-Site-Disordered Perovskite Oxide $CaLaScRuO_{6+\delta}$: An Efficient Oxygen Bifunctional Electrocatalyst in a Highly

Alkaline Medium. *ACS Appl. Mater. Interfaces* **12**, 9190–9200 (2020).

14. Retuerto, M. *et al.* Na-doped ruthenium perovskite electrocatalysts with improved oxygen evolution activity and durability in acidic media. *Nat. Commun.* **10**, 2041 (2019).
15. Sun, H. *et al.* Boosting oxygen evolution reaction activity of perovskite through introducing multi-elements synergy and building ordered structure. *J. Mater. Chem. A* **7**, 9924-9932 (2019).
16. Zhao, B. *et al.* A tailored double perovskite nanofiber catalyst enables ultrafast oxygen evolution. *Nat. Commun.* **8**, 14586 (2017).
17. Hua, B. *et al.* Stabilizing Double Perovskite for Effective Bifunctional Oxygen Electrocatalysis in Alkaline Conditions. *Chem. Mater.* **29**, 6228–6237 (2017).
18. Bian, J. *et al.* Mg doped perovskite $LaNiO_3$ nanofibers as an efficient bifunctional catalyst for rechargeable zinc-air batteries. *ACS Appl. Energy Mater.* **2**, 923–931 (2019).
19. Tong, Y. *et al.* Vibronic Superexchange in Double Perovskite Electrocatalyst for Efficient Electrocatalytic Oxygen Evolution. *J. Am. Chem. Soc.* **140**, 11165–11169 (2018).
20. Wang, H. *et al.* Polymer-assisted approach to $LaCo_{1-x}Ni_xO_3$ network nanostructures as bifunctional oxygen electrocatalysts. *Electrochim. Acta* **296**, 945–953 (2019).
21. Sun, Y. *et al.* Engineering of the d-Band Center of Perovskite Cobaltite for Enhanced Electrocatalytic Oxygen Evolution. *ChemSusChem* **13**, 2671–2676 (2020).
22. Chen, G. *et al.* Ultrahigh-performance tungsten-doped perovskites for the oxygen evolution reaction. *J. Mater. Chem. A* **6**, 9854–9859 (2018).
23. Zhu, K. *et al.* Oxygen evolution reaction over Fe site of $BaZrxFe_{1-x}O_{3-\delta}$ perovskite oxides. *Electrochim. Acta* **241**, 433–439 (2017).
24. Sun, H. *et al.* Molybdenum and Niobium Codoped B-Site-Ordered Double Perovskite Catalyst for Efficient Oxygen Evolution Reaction. *ACS Appl. Mater. Interfaces* **10**, 16939–16942 (2018).
25. Xu, X. *et al.* Co-doping strategy for developing perovskite oxides as highly efficient electrocatalysts for oxygen evolution reaction. *Adv. Sci.* **3**, 1500187 (2015).
26. Li, X. *et al.* Redox inactive ion meliorated $BaCo_{0.4}Fe_{0.4}Zr_{0.1}Y_{0.1}O_{3-\delta}$ perovskite oxides as efficient electrocatalysts for the oxygen evolution reaction. *J. Mater. Chem. A* **6**, 17288–17296 (2018).
27. Zhu, Y. *et al.* $SrNb_{0.1}Co_{0.7}Fe_{0.2}O_{3-\delta}$ Perovskite as a Next-Generation Electrocatalyst for Oxygen Evolution in Alkaline Solution . *Angew. Chemie* **127**, 3969–3973 (2015).
28. Liu, H. *et al.* Mixed protonic-electronic conducting perovskite oxide as a robust oxygen evolution reaction catalyst. *Electrochim. Acta* **282**, 324–330 (2018).
29. Sun, H. *et al.* Smart Control of Composition for Double Perovskite Electrocatalysts toward Enhanced Oxygen Evolution Reaction. *ChemSusChem* **12**, 5111–5116 (2019).

30. Su, C. *et al.* $SrCo_{0.9}Ti_{0.1}O_{3-\delta}$ As a New Electrocatalyst for the Oxygen Evolution Reaction in Alkaline Electrolyte with Stable Performance. *ACS Appl. Mater. Interfaces* **7**, 17663–17670 (2015).
31. Yang, L. *et al.* Enhanced Iridium Mass Activity of 6H-Phase, Ir-Based Perovskite with Nonprecious Incorporation for Acidic Oxygen Evolution Electrocatalysis. *ACS Appl. Mater. Interfaces* **11**, 42006–42013 (2019).
32. Gao, R. *et al.* Efficient acidic oxygen evolution reaction electrocatalyzed by iridium-based 12L-perovskites comprising trinuclear face-shared IrO_6 octahedral strings. *J. Energy Chem.* **47**, 291–298 (2020).
33. Zhang, R. *et al.* A Dissolution/Precipitation Equilibrium on the Surface of Iridium-Based Perovskites Controls Their Activity as Oxygen Evolution Reaction Catalysts in Acidic Media. *Angew. Chemie - Int. Ed.* **58**, 4571–4575 (2019).
34. Chen, Y. *et al.* Exceptionally active iridium evolved from a pseudo-cubic perovskite for oxygen evolution in acid. *Nat. Commun.* **10**, 572 (2019).
35. Miao, X. *et al.* Quadruple perovskite ruthenate as a highly efficient catalyst for acidic water oxidation. *Nat. Commun.* **10**, 3809 (2019).
36. Meng, J. *et al.* Advances in metal-organic framework coatings: Versatile synthesis and broad applications. *Chem. Soc. Rev.* **49**, 3142–3186 (2020).
37. Zhu, Y., Zhou, W., Sunarso, J., Zhong, Y. & Shao, Z. Phosphorus-Doped Perovskite Oxide as Highly Efficient Water Oxidation Electrocatalyst in Alkaline Solution. *Adv. Funct. Mater.* **26**, 5862–5872 (2016).
38. Du, X. *et al.* PLD-fabricated perovskite oxide nanofilm as efficient electrocatalyst with highly enhanced water oxidation performance. *Appl. Catal. B Environ.* **272**, 119046 (2020).
39. Xiong, J. *et al.* Engineering highly active oxygen sites in perovskite oxides for stable and efficient oxygen evolution. *Appl. Catal. B Environ.* **256**, 117817 (2019).
40. Hua, B. *et al.* Activating p-Blocking Centers in Perovskite for Efficient Water Splitting. *Chem* **4**, 2902–2916 (2018).
41. Wang, Y. *et al.* ScienceDirect Molecular-level proton acceptor boosts oxygen evolution catalysis to enable efficient industrial-scale water splitting. *Green Energy Environ.* (2022) DOI:10.1016/j.gee.2022.07.001.
42. She, S. *et al.* Realizing Ultrafast Oxygen Evolution by Introducing Proton Acceptor into Perovskites. *Adv. Energy Mater.* **9**, 1900429 (2019).
43. Curcio, A. *et al.* Unlocking the Potential of Mechanochemical Coupling: Boosting the Oxygen Evolution Reaction by Mating Proton Acceptors with Electron Donors. *Adv. Funct. Mater.* **31**, 2008077 (2021).
